# Triage of Atypical Glandular Cell by *SOX1* and *POU4F3* Methylation: A Taiwanese Gynecologic Oncology Group (TGOG) Study

**DOI:** 10.1371/journal.pone.0128705

**Published:** 2015-06-09

**Authors:** Cheng-Chang Chang, Yu-Che Ou, Kung-Liahng Wang, Ting-Chang Chang, Ya-Min Cheng, Chi-Hau Chen, Tang-Yuan Chu, Shih-Tien Hsu, Wen-Shiung Liou, Yin-Yi Chang, Hua-Hsi Wu, Tze-Ho Chen, Hung-Cheng Lai

**Affiliations:** 1 Department of Obstetrics and Gynecology, Tri-Service General Hospital, National Defense Medical Center, Taipei, Taiwan, R.O.C; 2 Graduate Institute of Medical Sciences, National Defense Medical Center, Taipei, Taiwan, R.O.C; 3 Department of Obstetrics and Gynecology, Kaohsiung Chang Gung Memorial Hospital and Chang Gung University College of Medicine, Kaohsiung, Taiwan, R.O.C; 4 Department of Obstetrics and Gynecology, Mackay Memorial Hospital, Taipei, Taiwan, R.O.C; 5 Department of Obstetrics and Gynecology, Linkou Chang Gung Memorial Hospital, Taoyuan, Taiwan, R.O.C; 6 Department of Obstetrics and Gynecology, National Cheng Kung University Hospital, Tainan, Taiwan, R.O.C; 7 Department of Obstetrics and Gynecology, National Taiwan University Hospital, Taipei, Taiwan, R.O.C; 8 Department of Obstetrics and Gynecology, Buddhist Tzu Chi General Hospital, Hualien, Taiwan, R.O.C; 9 Department of Obstetrics and Gynecology, Taichung Veterans General Hospital, Taichung, Taiwan, R.O.C; 10 Department of Obstetrics and Gynecology, Kaohsiung Veterans General Hospital, Kaohsiung, Taiwan, R.O.C; 11 Department of Obstetrics and Gynecology, China Medical University Hospital, Taichung, Taiwan, R.O.C; 12 Department of Obstetrics and Gynecology, Taipei Veterans General Hospital, Taipei, Taiwan, R.O.C; 13 Department of Obstetrics and Gynecology, Changhua Christian Hospital, Changhua, Taiwan, R.O.C; 14 Department of Obstetrics and Gynecology, Shuang Ho Hospital, Taipei Medical University, New Taipei City, Taiwan, R.O.C; 15 Department of Obstetrics and Gynecology, School of Medicine, College of Medicine, Taipei Medical University, Taipei, Taiwan, R.O.C; Istituto Nazionale Tumori, ITALY

## Abstract

**Introduction:**

Invasive procedures including loop electrosurgical excision, cervical conization, and endometrial sampling are often recommended when atypical glandular cells (AGC) are detected on Pap smear with unsatisfactory colposcopy. These invasive procedures may result in patient anxiety, increased medical expense, and increasing the risk of preterm delivery in subsequent pregnancies. This study was performed to assess methylation biomarkers in the triage of AGC on Pap smear for invasive procedures.

**Methods:**

We conducted a multicenter study in 13 medical centers in Taiwan from May 2012 to May 2014. A total of 55 samples diagnosed “AGC not otherwise specified” (AGC-NOS) were included. All patients with AGC underwent colposcopy, cervical biopsy, endometrial sampling, and conization if indicated. Multiplex quantitative methylation-specific polymerase chain reaction (QMSPCR) was performed. Sensitivity, specificity, and accuracy were calculated for detecting CIN3^+^ and endometrial complex hyperplasia.

**Results:**

In 55 patients with AGC, the sensitivity for methylated (^m^) *SOX1^m^*, *PAX1^ m^*, *ZNF582^m^,PTPRR^m^, AJAP1^m^, HS3ST2^m^,* and *POU4F3^m^* for detecting CIN3^+^ and endometrial complex hyperplasia lesions was 100, 86, 71, 86, 86, 57, and 100%; specificity was 67, 79, 85, 50, 52, 96, and 52%, respectively. Testing for high risk-HPV had a sensitivity of 57% and specificity of 75% for CIN3+ and endometrial complex hyperplasia lesions.

**Conclusion:**

Methylated (^m^) *SOX1^m ^*and *POU4F3^m ^*could be new methylation biomarkers for detection of CIN3^+^ and endometrial complex hyperplasia in AGC. Women with AGC and positive *SOX1^m ^*/ *POU4F3^m^*, colposcopy, cervical conization or endometrial sampling should be considered.

## Introduction

Since the introduction of cytological screening for cervical precancerous lesions several decades ago, there has been a significant reduction in the incidence of and mortality associated with cervical cancer [1]. Although the incidence of invasive squamous disease has decreased dramatically, there has been an increase in the relative and absolute incidence of cervical adenocarcinoma over the same period; cytological screening has not reduced the risk of adenocarcinoma as much as it has reduced the risk of squamous cell carcinoma [2–4].

The Pap smear has a lower sensitivity in diagnosing glandular lesions. Atypical glandular cells (AGC) constitute a rare cytological category, with <0.5% of all Pap tests designated as having such cells [5, 6]. An AGC interpretation is reproduced poorly between observers [7]. The management of AGC remained unsolved. Despite endometrial sampling and cervical conization are suggested by American society for colposcopy and cervical pathology (ASCCP) if colposcopy is negative. Although the crude incidence of subsequent squamous cell carcinoma and adenocarcinoma could be as high as 262.9 (95% CI, 200.4–325.4) and 174.0 (95% CI, 123.1–224.8) per 100,000 person-years, respectively [2], only 11.1%, 2.9% and 5.2% were diagnosed as high-grade intraepithelial lesion (HSIL), adenocarcinoma in situ(AIS) and invasive cancer(including endometrium, cervix, ovary and fallopian tube), respectively [6]. Therefore, most women with AGC are over treated.

HPV plays an essential role in cervical carcinogenesis. The detection of high-risk HPV shows a sensitivity of 60% to 96% (with low specificity) in the prediction of significant pathological findings of AGC on Pap smear [8–10]. Risk for high grade lesions including adenocarcinoma of cervix or endometrium [6], which is not possible to be detected by colposcopy. HPV testing was reported as a useful biomarker for the triage of atypical squamous cell (ASC). However, the role of HPV testing in the triage of AGC is unsatisfactory. A balance between the detection of occult disease and overtreatment is called for because concerns about undetected endocervical disease have resulted in a high frequency of negative excisional biopsies. Findings were benign in 68.5–71% of women who tested positive for high-risk HPV [6, 11], including 61.4% of those who had undergone excisional procedures [11]. Alternative strategies, including endometrial sampling, human papillomavirus testing, and cervical conization should be considered if colposcopy is negative. Overtreatment may result in patient anxiety, increased medical expenses, and cervical incompetence with resultant preterm delivery in subsequent pregnancies. A better biomarker for triage of AGC is needed.

In comparing normal, precancerous, and cervical cancer tissue, the DNA methylation signatures of the host genome may identify tissue-specific perturbations that occur during carcinogenesis [12]. Methylation leaves a heritable record of such interactions and is an ideal biomarker for cancer detection [13–17]. Therefore, DNA methylation could be used as a biomarker for cervical cancer detection [18–21]. Previously, we used a differential methylation hybridization method and identified novel genes including *PAX1* and *SOX1* that were silenced by methylation in cervical SCCs [14]. Quantitative analysis of PAX1 and SOX1 genes is effective in the detection of cervical intraepithelial neoplasia (CIN) grade 3, or worse [15]. By other two methylomic methods, we further discovered Zinc finger protein 582 (*ZNF582*), *AJAP1*, *HS3ST2*, and *POU4F3* are highly methylated in cancer and precancerous lesions [16, 22]. *PAX1*, *PTPRR*, *SOX1*, and *ZNF-582* genes are also highly methylated in adenocarcinoma (AC) of the cervix [23]. In our previous work using a methylation biomarker in the triage of abnormal Pap smears, we show that *PAX1* high methylation analysis may be a better choice than HC2 in the triage of ASC and ASC-H [24]. In a Taiwan Gynecology Oncology Group (TGOG) study, we used a methylation biomarker and H-HPV to detect CIN3+ lesions in low-grade squamous intraepithelial lesions (LSIL). *ZNF582* methylation analysis in cervical scrapings may be a promising biomarker in the positive triage of cytological diagnoses of LSIL [22]. High methylation of *SOX1* can also serve as a biomarker for hidden cancer in endometrial atypical hyperplasia (manuscript accepted for publication). All there evidences suggest the potential role of DNA methylation in the triage of AGC. There are no reports in the literature so far.

We conducted a multicenter study in 13 medical centers in Taiwan to assess methylation biomarkers (*SOX1*, *PAX1*, *ZNF582*, *PTPRR*, *AJAP1*, *HS3ST2*, *POU4F3*, and *ADRA1D* genes) to detect CIN3+/CIS and complex hyperplasia of the endometrium in AGC identified on Pap smear for invasive procedures.

## Materials and Methods

### Patients

**Fig 1:**
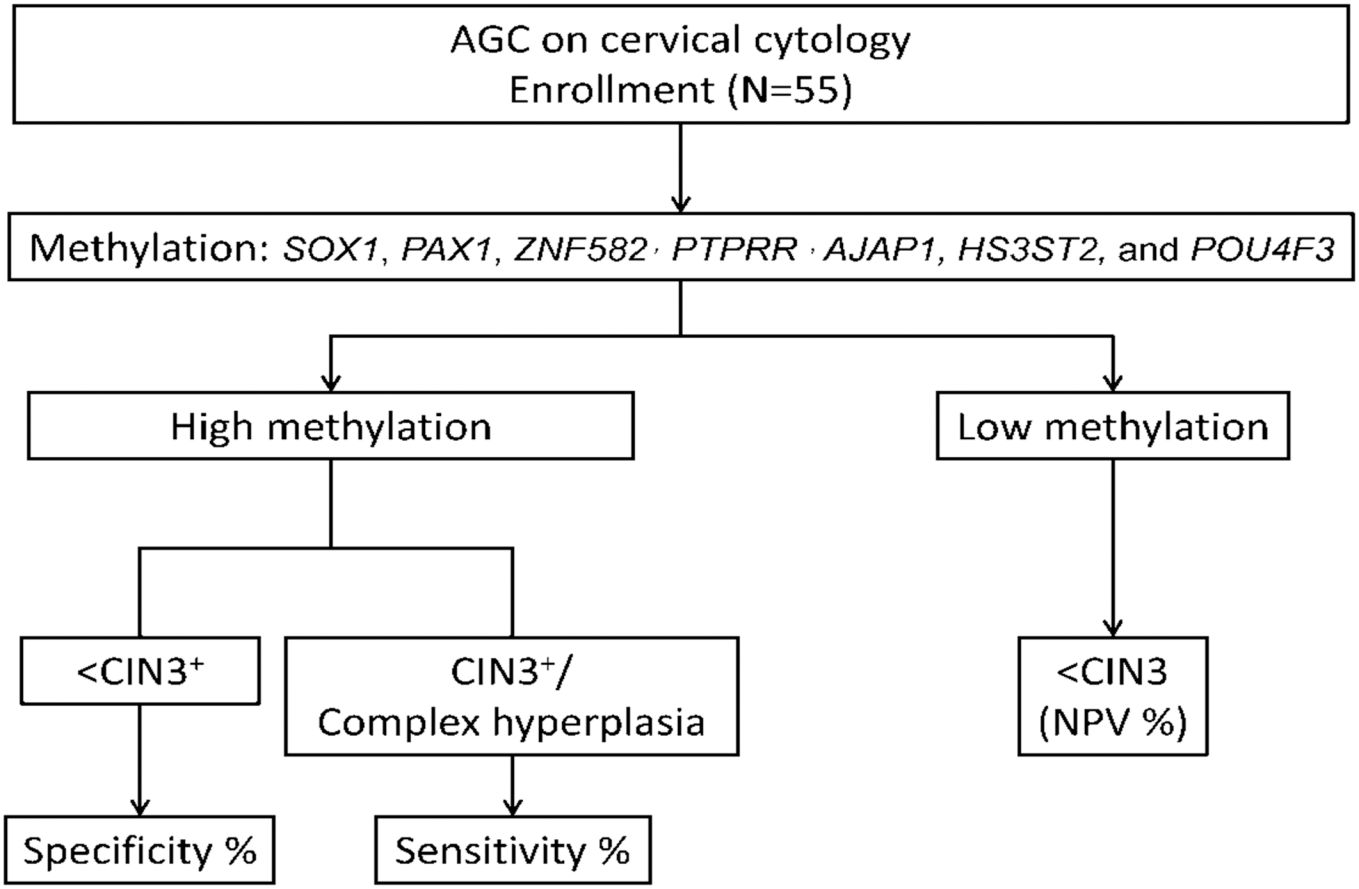
Study flowchart: All women were invited to participate in diagnosis of AGC with a methylation biomarker study. Physician-directed sampling was performed with a cytobrush (CooperSurgical, Inc.). Patients with Pap smear of AGC underwent colposcopy and cervical biopsy, endometrial sampling, or conization in accordance with treatment guidelines. CIN III/CIS and complex hyperplasia of the endometrium were taken as cutoff criteria for QMS-PCR of seven genes. The receiver operating characteristic (ROC) curve was used to select the optimal cutoff value for the methylation level of each gene. Methylation levels that were higher than the threshold were classified as “high methylation”, whereas those that were lower than the threshold were recorded as “low methylation”.

We conducted a multicenter study in 13 medical centers in Taiwan from May 2012 to May 2014. A total of 55 samples diagnosed “AGC not otherwise specified” (AGC-NOS) were included. The Study Flowchart is shown in Fig 1. All AGC was managed with colposcopy, cervical biopsy, endometrial sampling, and LEEP/conization if indicated. There were 4 cervical intraepithelial neoplasm, grade 3(CIN3)/carcinoma in situ (CIS), 1 squamous cell carcinoma (SCC), 1 adenocarcinoma (AC) and 1 complex hyperplasia of endometrium confirmed by histopathology.

### Ethics statement

All participants provide their written informed consent to participate in this study. This study was conducted in accordance with the guidelines, and with the approval of, the Ethics Committee of the Institutional Review Boards of the Tri-Service General Hospital, National Defense Medical Center (TSGHNDMCIRB-096-05-090).

### HPV testing

Infection with HR-HPV was detected using Hybrid Capture 2 (HC2) test kits (Digene, Silver Spring, MD) according to the manufacturer’s protocol, which can detect HPV types 16, 18, 31, 33, 35, 39, 45, 51, 52, 56, 58, 59, and 68. Samples with a relative light units/cutoff value ratio higher than 1.0 were recorded as positive.

### Amplification of DNA with real-time quantitative methylation-specific polymerase chain reaction (QMSP)

Genomic DNA was extracted from the collected specimens by using an established protocol for tissue banking. The concentration of DNA was determined by using the Nanodrop 1000 (Thermo Fisher Scientific Inc). QMSP was performed after bisulfite treatment of denatured genomic DNA. Primers and probes for QMSP were published previously.[15, 22, 25]The COL2A gene was used as an internal reference to adjust the amount of input DN by amplifying non CpG sequences in each sample. QMSP was performed with a TaqMan probe system in Roche LightCycler 480 system. The 5’ and 3’ ends of the probes were separately labeled with 6-carboxy-fluorescein (6-FAM) and with a quencher dye. The 20 μL reaction contained 2 μL of bisulfite template DNA, 250nM of each primer, 225nM of TaqMan probe, and 10 μL of FastStart Universal Probe Master (ROX, Roche). For the TaqMan-based QMSP, each sample was analyzed in duplicate. The reactions were performed by using an initial incubation at 95°C for 10 min, followed by 45 cycles of 95°C for 15 s and annealing and extension at the appropriate temperatures for 1 min. The level of DNA methylation was described as the methylation index (M-index): 10,000 × 2[(Cp of COL2A)–(Cp of Gene)] [16]. The QMSP was ruled a failure if the Cp value of *COL2A* was higher than 36.

### Statistics analysis

The data were analyzed by using statistical package SPSS version 20.0 for Windows (IBM Corp., Armonk, NY, USA). To determine the detection rate, CIN 3^+^/CIS and complex hyperplasia of endometrium were taken as cutoff criteria for the QMS-PCR of seven genes in sampling. The receiver operating characteristic (ROC) curve was used to select the optimal cutoff value for the methylation level of each gene. Methylation levels that were higher than the threshold were classified as “high methylation”, whereas those that were lower than the threshold were recorded as “low methylation” in study samples. Sensitivity and specificity were calculated for detection of CIN3+ (including CIN3, CIS, SCC, AC and complex hyperplasia). Methylation rate and statistical power were calculated by STATA 9. Trend test was calculated by Kendall’s Tau correlation = τ.

## Results

### The histopathology and HPV results of AGC patients

We enrolled 55 patients whose Pap smear showed AGC (Table 1). The mean age of the enrolled patients was 48 (24–85). In seven patients (12.7%) histology demonstrated significant cervical disease (lesions of CIN3 or worse) or endometrial lesions (complex hyperplasia), included 4 cervical intraepithelial neoplasm, grade 3(CIN3)/carcinoma in situ (CIS), 1squamous cell carcinoma (SCC), 1 adenocarcinoma (AC) and 1 complex hyperplasia of endometrium. No abnormality was detected in 31 patients (56.4%), and minor cervical lesions (CIN1) were detected in 12 patients (21.8%). High-risk HPV was detected in 16 patients (29.1%) (Table 1).

**Table 1:** Pathology and HPV results of AGC patients.

Age	Mean	(Range)
	48	(24–85)
Results of Pathology	No	(%)
Normal	31	(56.4)
CIN 1	12	(21.8)
CIN 2	3	(5.5)
CIN 3/CIS	4	(7.3)
SCC	1	(1.8)
AC	1	(1.8)
Simple hyperplasia	2	(3.6)
Complex hyperplasia	1	(1.8)
HPV typing	No	(%)
Negative	39	(70.9)
Positive	16	(29.1)
Total number	55	(100)

Abbreviations: AGC, atypical glandular cell; CIN1, cervical intraepithelial neoplasia type 1, CIN2, cervical intraepithelial neoplasia type 2; CIN3, cervical intraepithelial neoplasia type 3; CIS, carcinoma in situ; SCC, squamous cervical carcinoma; AC, adenocarcinoma.

### Methylation index of *SOX1*, *PAX1*, *ZNF582*, *PTPRR*, *AJAP1*, *HS3ST2*, *POU4F3*, and *ADRA1D* genes

**Fig 2:**
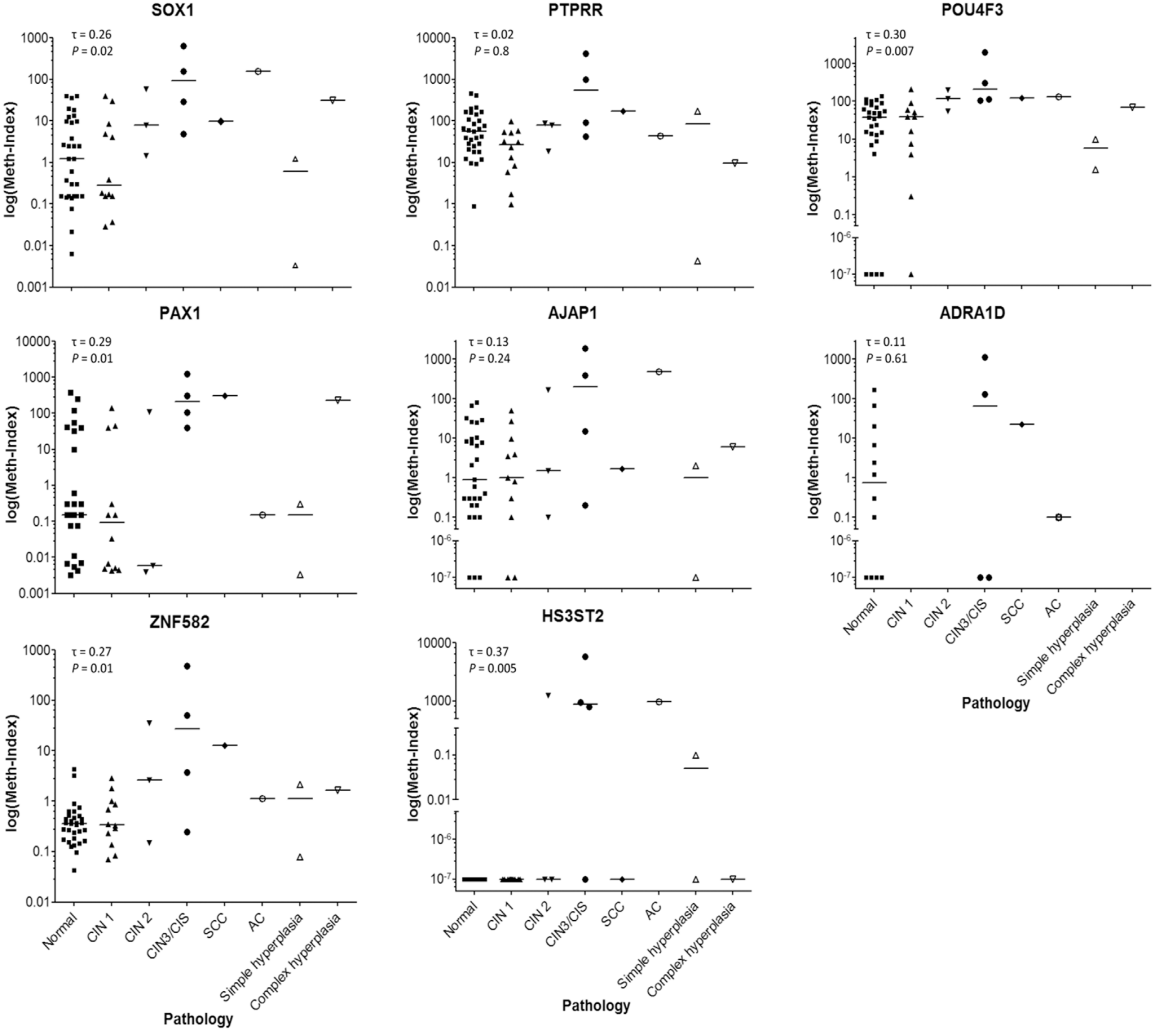
Methylation index (meth-index) on a log scale of methylated (^m^) *SOX1* ^*m*^, *PAX* ^*m*^, *ZNF582* ^*m*^, *PTPRR* ^*m*^,*AJAP1* ^*m*^, *HS3ST2* ^*m*^, and *POU4F3* ^*m*^ levels from scrapings of the normal cervix and tumors graded as CIN1, CIN2, CIN3, CIS, or SCC/AC, or endometrial lesions (including simple hyperplasia and complex hyperplasia of the endometrium) by histopathology. Each dot represents the testing result of one patient.

We analyzed the methylation status of *SOX1* ^*m*^, *PAX1* ^*m*^, *ZNF582* ^*m*^, *PTPRR* ^*m*^, *AJAP1* ^*m*^, *HS3ST2* ^*m*^, *POU4F3* ^*m*^, and *ADRA1D* ^*m*^ genes in AGC scrapings. Methylation index (meth-index) was measured on a log scale for methylated (^m^) *SOX1* ^*m*^, *PAX1* ^*m*^, *ZNF582* ^*m*^, *PTPRR* ^*m*^, *AJAP1* ^*m*^, *HS3ST2* ^*m*^, and *POU4F3* ^*m*^ levels from scrapings of the normal cervix and tumors graded as CIN1, CIN2, CIN3, CIS, SCC/AC, or endometrial lesions (including simple hyperplasia and complex hyperplasia of the endometrium) by histopathology (Fig 2). Each dot represents the test results of one patient. The trend of methylation status increased progressively from normal (lowest methylation), CIN1, CIN2, CIN3, CIS, to SCC/AC (highest methylation) in the genes *SOX1*, *PAX1*, *ZNF582*, *HS3ST2* and *POU4F3* with statistical significance (test for trend was calculated by Kendall’s Tau correlation = τ, P<0.05) (Fig 2). The methylation status of complex hyperplasia was also higher than that of simple hyperplasia in *SOX1*, *PAX1*, ZNF582, AJAP1 and *POU4F3* (Fig 2).

### The clinical accuracy of *SOX1*, *PAX1*, *ZNF582*, *PTPRR*, *AJAP1*, *HS3ST2*, *POU4F3*, and *ADRA1D* genes in the triage of AGC patients

Sensitivity and specificity were calculated for detection of CIN3+ (including CIN3, CIS, SCC, AC and complex hyperplasia). The clinical accuracy by methylation biomarkers of *SOX1*, *PAX1*, *ZNF582*, *PTPRR*, *AJAP1*, *HS3ST2*, *POU4F3*, and *ADRA1D* in AGC were shown in Table 2. All cases of significant cervical disease (CIN3+ /CIS or worse lesions) and complex hyperplasia of the endometrium were detected by methylation markers *SOX1*and *POU4F3*, with a sensitivity of 100% (95% CI,59–100) and specificity of 67% (95% CI,52–80) and 52% (95% CI,37–67), respectively (Table 2). *SOX1*has the highest AUC as 0.83(95% CI, 0.72–0.94).

**Table 2:** Clinical performance of methylation biomarker and HPV to detect CIN3^+^ /CIS and complex hyperplasia of endometrium in AGC.

Testing	M-Index^1^ cut-off value	Sen.(%, 95% CI)	Spe.(%, 95% CI)	AUC^2^
HPV test	none	57 (18–90)	75 (60–86)	0.66 (0.43–0.89)
SOX1	3	100(59–100)	67 (52–80)	0.83 (0.72–0.94)
PAX1	29	86 (42–100)	79 (65–90)	0.82 (0.66–0.99)
ZNF582	1.6	71(29–96)	85 (72–94)	0.78 (0.58–0.99)
PTPRR	40	86 (42–100)	50 (35–65)	0.68 (0.49–0.87)
AJAP1	1.6	86 (42–100)	52 (37–67)	0.69 (0.50–0.88)
HS3ST2	240	57 (18–90)	96 (85–99)	0.76 (0.53–1.00)
POU4F3	40	100(59–100)	52 (37–67)	0.76 (0.62–0.90)
ADRA1D	20	50 (12–88)	83 (52–98)	0.67 (0.38–0.95)

Abbreviations: AGC, atypical glandular cell; CIN3, cervical intraepithelial neoplasia type 3. The case number of AGC is 55, and the case number of CIN3+ is 7.

^1^Methylation-Index (M-Index): the value cut off for precancer/cancer patient (cervical intraepithelial neoplasia grade 3 and worse, CIN3+) or not (CIN2-, including normal specimens, and CIN1 and CIN2 specimens).

^2^The area under the ROC curve (AUC) with 95% confidence interval (CI) of each gene was calculated for the diagnosis of CIN3+ compare with control (CIN2-).

## Discussion

To fill up the gap of unsatisfactory role of Pap smear and HPV in the triage of AGC, our study demonstrated that the sensitivity of methylated *SOX1* and *POU4F3* in the detection of CIN3+ lesions and complex hyperplasia of endometrium is 100% with acceptable specificity, which may reduce about half invasive procedures in women with AGC. Because of the high sensitivity of methylation testing to detect significant cervical disease, patients with a positive methylation test result may be instructed to return for colposcopy, and those with a negative methylation test result may receive routine follow-up care. To our knowledge, this is the first report demonstrating the clinical potential of a methylation biomarker in the triage of AGC.

Detection of AGC was most often associated with neoplastic cervical lesions in women younger than 40 years of age, and with neoplastic endometrial lesions in women 50 years of age or older. HPV co-testing would not have aided screening in the detection the malignancies of endometrial origin diagnosed after AGC Pap results [5]. A large-scale study by Castle et al. demonstrated that HPV test results for women with AGC cytology may not be useful for triage. Additional biomarkers must be found for use in combination with HC2 H-HPV or replace HPV testing to reduce false-positive rates [26]. The combination of carbonic anhydrase-IX (CA-IX) and H-HPV testing had a sensitivity of 97% for significant cervical lesions (reflecting the presence of CIN2, CIN3, AIS, or invasive carcinoma), 100% for glandular lesions (adenocarcinoma in situ and adenocarcinoma), and 93% for significant squamous lesions (CIN2, CIN3, or squamous cell carcinoma) in Japanese women [27]. However, the results could not be validated in the United States [28]. These studies did not evaluate endometrial pathology which is an important concern when dealing with women with AGC, when age of more than 50. The sensitivity, specificity, PPV, and NPV for CA-IX vs SOX1 and POU4F3 are summarized in Table 3. Our results also successfully detected the only one complex hyperplasia of endometrium. Although there is good evidence that p16 immunostaining correlates with severity of cytological/histological abnormalities, the reproducibility is limited due to insufficient standardization of interpretation of the immunostaining [29]. Taken together, *SOX1* ^(m)^ or *POU4F3* ^(m)^ are promising biomarkers for the triage of AGC. A randomized trial is planned by TGOG to validate this algorithm, which may change the current guidance of clinical practice in the future.

**Table 3:** Summary of clinical performance of methylation biomarker, HPV and CA-IX.

Testing	Sensitivity	Specificity	PPV	NPV
SOX1	100	67	30	100
POU4F3	100	52	24	100
HPV(HC2)	57	75	25	92
CA-IX* ^,^ ^a^	75	88	70	90
CA-IX** ^,^ ^a^	89	47	55	86
HPV(HC2)* ^,^ ^a^	97	87	77	99
HPV(PCR)** ^,^ ^a^	65	86	76	78
HPV+CA-IX* ^,^ ^a^	97	80	69	99
HPV+CA-IX** ^,^ ^a^	97	42	53	95

Abbreviations: HPV, human papilloma virus; CA-IX, carbonic anhydrase-IX.

^a^The studies is only using significant cervical lesion, reflects the presence of CIN2, CIN3, AIS or invasive carcinoma.

*Int. J. Cancer: 125, 2434–2440 (2009)

**British Journal of Cancer 104, 353–360 (2011)

The risk of CIN3+ and cancer when AGC was detected were 12.7% and 3.6% reporting in the present study, which is much higher than the reports from a larger study by Castle et al (8.2% and 1.7%, respectively) [30]. These data can be inferred that Pap smear were underestimated and that they could be classified as AGC = NOS, should be classified as AGC-FN, or suggesting the stringent cytology quality control in Taiwan. No abnormality was detected in 56.4% of participants who had undergone excisional procedures, and minor cervical lesions (CIN1) were detected in 21.8%. Therefore, 78.2% of participants with AGC may have undergone unnecessary invasive procedures. The application of *SOX1*/*POU4F3* methylation may reduce more invasive procedures in AGC women in USA or countries with unsatisfactory cytology quality control. We used CIN3+ rather than CIN2+ as the cutoff in our study because of the equivocal nature of diagnosis of CIN2 lesions, and the heterogeneity of their DNA methylation profiles [15, 31]. Whereas only 5% of CIN2 lesions progress to invasive cancer, and approximately 40% regress, the corresponding percentages for CIN3 lesions are 33% and 12%, respectively [1]. CIN2 lesions are a gray zone in pathology and are the most difficult for pathologists to confirm among all Pap smear diagnoses [32, 33]. CIN2 is regarded as an in-between diagnosis that could be over-called CIN1 or under-called CIN3. In fact, the natural history of CIN2 tumors is different from that of CIN3 lesions [31]. Therefore, the clinical management of patients with CIN2 lesions should be reassessed with the most accurate techniques. The incorporation of molecular markers in cervical cancer screening, such as DNA methylation markers, might help to decrease the number of unnecessary referrals and repeat diagnostic procedures, which not only waste money but also inflict a needless burden on the patient. Additional studies are required to define the screening interval for use of DNA methylation biomarkers to diagnose these patients properly.

The function of SOX1 and POU4F3 in cancer biology remain largely unknown. The SOX1proteinis a transcription factor important in developmental processes [34]. The expression of *SOX1* attenuates the tumorigenic potential of neuronal precursors after neural stem cell transplantation, suggesting a role in stem cell differentiation [35]. A recent study showed that *SOX1* can be a tumor suppressor partly through the Wnt/β-catenin signaling pathway in cervical cancer [25]. DNA methylation analysis of *SOX1* and *POU4F3* in cervical scrapings consistently detects cervical cancer and the majority of CIN3 lesions, and has the capacity to broaden its use on cervical scrapings through the detection of a substantial subset of complex hyperplasia. We use the sample size included 7 cases of CIN3+ (including CIN3, CIS, SCC, AC and complex hyperplasia) and 48 cases of CIN2^-^ (including normal controls, CIN1, CIN2 and simple hyperplasia) to calculate the sufficient power of the study. The statistical power was calculated under type 1 error rate of 0.05, two-sided, and the methylation rate of SOX1 in CIN3^+^ was 100%, in CIN3^-^ was 33.3%, the statistical power was 99.8%. The methylation rate of POU4F3 in CIN3^+^ was 100%, in CIN3^-^ was 45.8%, the statistical power was 81.4%. Methylation rate and statistical power were calculated by STATA 9.

**Fig 3:**
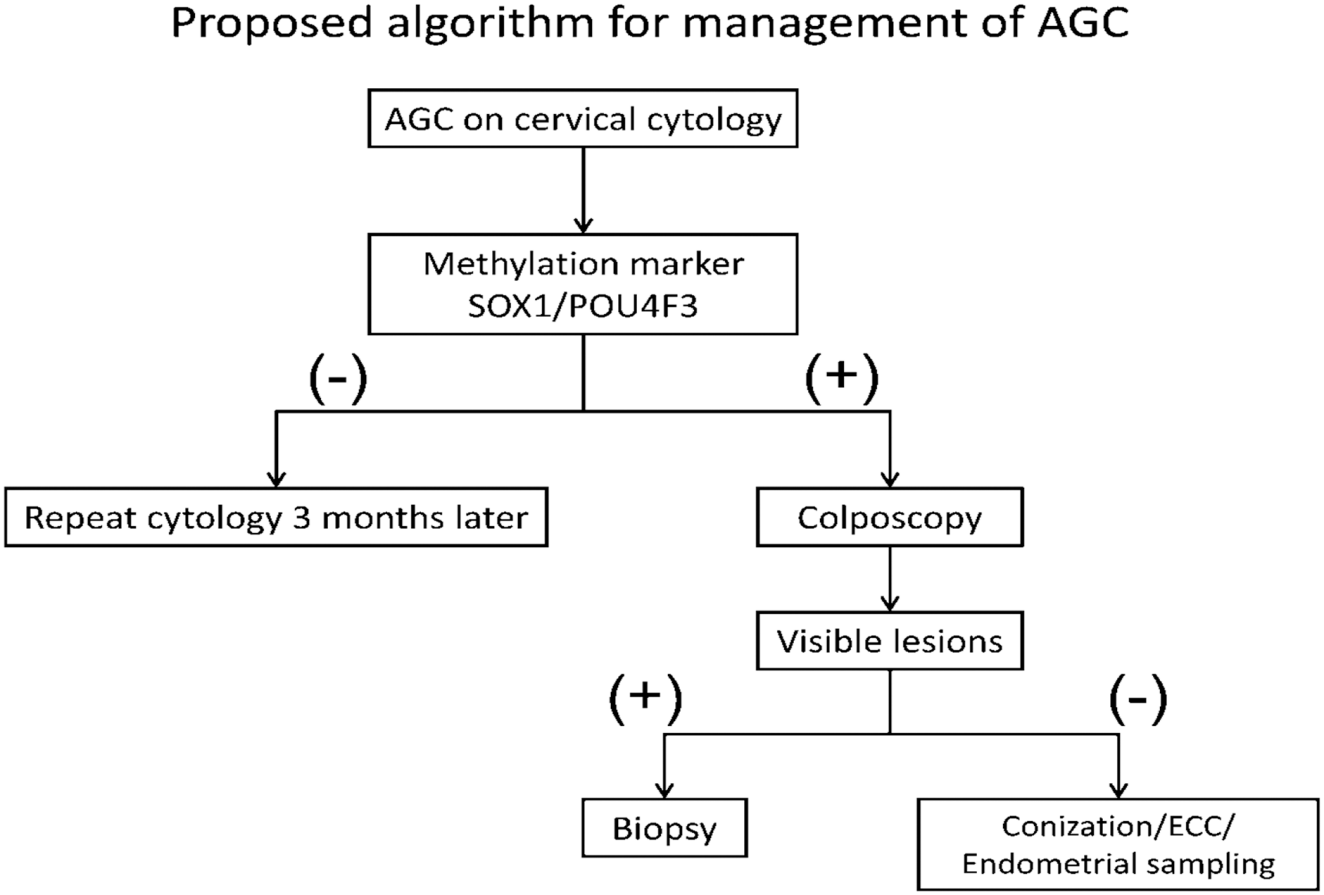
Proposed algorithm for management of AGC in TGOG study. For women with cytology identifying AGC, methylation biomarkers of *SOX1* and *POU4F3* are recommended. If, after a comprehensive examination, women with positive *SOX1* ^***m***^ and *POU4F3* ^***m***^ have no recognizable disease, a cervical conization or an endometrial sampling may be considered, otherwise, repeat cytology 3 months later should be the preferred recommendation because the sensitivity of *SOX1* and *POU4F3* is 100%.

This is a multi-center clinical study to demonstrate that methylation biomarker for detection of true lesions in atypical glandular cell of Pap smear is feasible. Base on the result and statistical power, they would provide evidence in support of the use of these new biomarkers as a new triage tool for cervical cancer detection in the future. In consideration of our findings, we would like to propose an algorithm for triage of AGC (Fig 3): for women with AGC noted on cytology and positive *SOX1* ^*m*^ and *POU4F3* ^*m*^, colposcopic biopsy with no identified lesion, a cervical conization or endometrial sampling may be considered.

## Conclusion

Methylated (^m^) *SOX1* ^*m*^ and *POU4F3* ^*m*^ are potential new biomarkers for detection of CIN3^+^ and complex hyperplasia lesions in AGC. Women with AGC and positive *SOX1* ^*m*^ or *POU4F3* ^*m*^ should go for colposcopy, conization or endometrial sampling. A randomized trial is on planning by TGOG.
